# Winter Quarters

**Published:** 1897-11-13

**Authors:** 


### WINTER QUARTERS.

Invalids have been fortunate this year in the fact that winter has not come early, and that during October, and even the beginning of November, they have been able to enjoy a fair modicum of sunshine. But this must end, winter must come, and even as we write signs are not lacking that the dread season is upon us. Invalids will now find their vigour put to the test, convalescents will now see whether their recovery has or has not been complete, children will have to stand either the strain of cold or the deteriorating influence of confinement, and old people will have to face the question, which to each individual becomes more serious and more anxious as each year passes by, whether they will be able, yet once again, to live through the winter.

The question then of winter quarters becomes one of great interest, and it may be added of no small anxiety, for once fixed it is difficult for the real invalid to make a change, and wherever he finds himself placed he usually has to make the best of it until the genial warmth of spring again unlocks the roads. It cannot be denied that the winter journey is a danger to which many sick people succumb every year, and that, however those who travel on pleasure bent may act, those who seek for health in sunny climes should get there while the journey can be made in comfort.

It is not to be wondered at that, knowing how near are the bright and sunny shores of the Riviera, many a sick man in England should turn restive, and should demand at all hazard to be transported from northern fogs and carried to places where such life as still remains can be enjoyed. But it cannot too strongly impressed on the would-be traveller that the place for the real invalid is home. To those who possess wealth, and are prepared to spend it, who can carry with them their home comforts and many of their home surroundings, nothing is better than to run away from snow and fogs and seek the sun. But it is otherwise with those who have to scrape and pinch, who have to put up with second-rate accommodation, who cannot provide themselves with the comforts to which at home they are accustomed, and are day by day worried and depressed by constant calls upon an ill-filled purse. Bad as the climate of England may be, it is often better to put up even with it than to face the discomforts of cheap travel, which, however trifling and even enjoyable they may seem in healthy youth, are full of danger to the invalid.

The cases, indeed, which gain the most striking benefit from wintering in the south are often hose which neither to the patient nor the riends seem serious enough to make such an excursion necessary. To all who are really ill there are two sides to the question of a journey southwards. There is both the profit and the loss, an undoubted gain in many cases, but often a certain risk. But to those who are merely suffering from debility, from slow convalescence, or from that growing susceptibility to climatic influences which so often comes with advancing age, there is practically no other side to the account, and the winter in the south is an unmitigated good.

Then come the whole tribe of kidney diseases and of cases of bronchitis and imperfect hearts, often interchangeable terms, together with many cases of more or less chronic dyspepsia—people who can keep well as long as they are out of doors, but fail as soon as they are shut up. The sun is to them the giver of all good. Time was when a winter on the Riviera was the great resort for persons suffering from phthisis, and there is no doubt that many so affected are enabled to live on by passing their winters in one or other of the sheltered spots to be found along that coast between the mountains and the sea. But in early phthisis we have now better means of cure than are offered by the health resorts of southern Europe. There can be but little doubt that the treatment by residence at high altitudes is, taking it all round, far more beneficial in such cases than any other method. Davos, St. Moritz, Arosa, and other places in Switzerland, besides many places in the Rocky Mountains, are available for this purpose. It has to be recognised, however, that phthisis is beginning to be looked upon more and more as a disease apart, and as one to be treated by itself. The knowledge that tuberculosis is an infective disorder has become so widely diffused that consumptive patients are now looked at very shyly by hotel keepers and others who, time ago, would have received them with open arms, and there can hardly be a question that this disease will in the future be largely treated in special sanatoria devised and managed with the special view of not only supplying the proper régime, but of preventing the diffusion of the infection. There is a growing feeling that some of the hotels in places which are largely resorted to by consumptives are not altogether free from danger as centres of infection, and great as have been the benefits to be derived from residence at high altitudes, it is by no means improbable that they will in the future be greater still when proper sanatoria are erected. In any case it must be remembered that climates are not to be judged by their efficacy in the treatment of consumption.

